# Proximal femoral replacement for oncologic and non-oncologic indications: a retrospective study over a 13-year period

**DOI:** 10.1007/s00068-026-03151-2

**Published:** 2026-03-31

**Authors:** Ricardo Ramón, Aktas Gökmen, Jorge Mayor, Hür Özbek, Adrian Niemann, Luis Marín, Tarek Omar Pacha, Mohamed Omar, Marco Ezechieli, Tilman Graulich

**Affiliations:** 1 Department of Trauma Surgery, Hannover Medical School, Hanover, Germany; 2 University of the North, Barranquilla, Colombia; 3 Clinic for Orthopedic Surgery ATOS Fleetinsel Hamburg, Hamburg, Germany; 4 Department for Orthopedic Surgery and Trauma in Pius-Hospital Oldenburg, Oldenburg, Germany

**Keywords:** Proximal femoral replacement, Infection, Tumor, Limb-salvage, Revision arthroplasty

## Abstract

**Background:**

Proximal femoral replacement (PFR) is a critical limb-salvage strategy for managing massive proximal femoral bone loss due to oncologic and non-oncologic conditions. Although oncologic outcomes are well described, evidence surrounding PFR for infection, periprosthetic fracture, and mechanical failure remains limited. This study evaluates short- to mid-term outcomes following PFR for multiple indications and compares primary with revision procedures.

**Methods:**

We retrospectively reviewed all patients undergoing PFR at a Level I trauma center from 2009 to 2023. Cases were categorized by procedure type (primary vs. revision) and indication (tumor, infection, other causes). Outcomes included operative time, complications according to the Henderson classification, reoperation rates, and functional outcomes.

**Results:**

A total of 81 patients (84 procedures) were included with a median follow-up of 7 months (IQR 0–31); 57.1% were lost to follow-up. The overall complication rate was 42.9%, significantly higher in revision procedures than in primary PFRs. Infection (Henderson Type IV) was the most frequent complication and occurred predominantly in revision surgeries. No cases of aseptic loosening (Type II) were identified. Reoperation was required in 29% of all procedures, with the highest rates observed in infection cases.

**Conclusions:**

PFR remains a reliable limb-salvage option for extensive proximal femoral deficiency. Revision procedures, especially infection-related cases, carry substantially higher risks of complications and reoperation. In contrast, primary oncologic PFRs showed comparatively favorable outcomes. Infection remains the principal barrier to durable results, underscoring the need for optimized perioperative strategies.

**Level of evidence:**

Therapeutic Level III (retrospective cohort study).

**Supplementary Information:**

The online version contains supplementary material available at 10.1007/s00068-026-03151-2.

## Introduction

Proximal Femoral replacement (PFR) is an established limb-salvage procedure used to treat massive proximal femoral defects resulting from primary bone tumors, metastatic disease, periprosthetic fractures, and complex revision arthroplasty [1–4]. Megaprosthetic reconstruction enables immediate stability and weight-bearing in situations where conventional reconstruction is not feasible, and the development of modular systems has expanded these applications in both oncologic and non-oncologic settings [4–7]. At our institutions, PFRs are predominantly performed using the MUTARS^®^ modular system, which is widely used in tumor and revision surgery and has demonstrated reliability across diverse indications [8–11].

The proximal femur is one of the most commonly affected sites for both primary malignant bone tumors and metastatic lesions, making it a key target for limb-salvage procedures [8, 12]. Although PFR was originally developed for oncologic management, its use has broadened significantly to include periprosthetic joint infection (PJI), periprosthetic fracture, implant failure, and aseptic loosening [3, 5–7, 10, 13–17].

These non-oncologic indications, however, carry distinct risk profiles, particularly regarding infection, soft-tissue compromise, and the cumulative burden of prior surgeries. Immediate weight bearing and reliable mechanical stability have helped establish PFR as a practical and effective reconstructive option in such complex scenarios [5, 6, 10, 12, 15, 18–20].

Given the heterogeneity of indications and the limited evidence comparing oncologic and non-oncologic outcomes, the present study evaluates short- to mid-term results of PFR in tumor resection, infection, and other mechanical causes, with additional comparison of outcomes between primary and revision procedures.

The study aimed to evaluate short- to mid-term outcomes following PFR as a limb-salvage reconstructive strategy at a single Level I trauma center across multiple indications, including tumor resection, infection and other mechanical causes. Outcomes were compared between primary and revision PFR procedures. The primary endpoint was any complication requiring surgical revision, while secondary endpoints included overall complication rate, Henderson failure mode distribution, operative duration, intensive care unit requirement, reoperation rate and mortality. We hypothesized that revision PFRs, particularly those performed because of infection, would be associated with significantly higher complication and reoperation rates compared with primary PFRs performed for oncologic indications.

## Materials and methods

This retrospective study included all patients who underwent proximal femoral replacement at our Level I trauma center between October 2009 and August 2023. Electronic medical records allowed consistent retrieval of clinical, operative, and follow-up information throughout the study period. Patients were eligible if they received a PFR either as primary treatment for bone or soft-tissue tumors or as revision surgery for periprosthetic joint infection, aseptic loosening, mechanical failure, or failed internal fixation. Only procedures performed using the MUTARS^®^ system were included. This resulted in a final cohort of 81 patients who underwent 84 proximal femoral replacement procedures during the study period, three patients underwent bilateral procedures; analyses were performed at the procedure level unless otherwise specified. Each case was categorized as either a primary or a revision procedure, and further stratified according to surgical indication into tumor, infection, or other causes such as periprosthetic fracture or mechanical complications.

Data were extracted from operative reports, inpatient notes, radiographic archives, and discharge summaries. Patients or relatives were contacted by telephone or mail to obtain updated follow-up and long-term outcomes. Variables collected included demographic characteristics, comorbidities, operative time, perioperative course including intensive care admission, complications, reoperations, mortality, and functional outcomes. Follow-up intervals were defined according to a modified framework from Ahmad et al., distinguishing short-term (≤ 3 months), mid-term (3 months to 2 years), and long-term (> 2 years) follow-up [21]. We use the terms short- and mid-term to describe early postoperative periods in this retrospective cohort. Given the short overall follow-up and substantial loss to follow-up, durability beyond two years cannot be inferred from most cases.

Complications were recorded according to the Henderson Classification **(**Table 1**)**, which defines five modes of failure: Type I (soft-tissue failure), Type II (aseptic loosening), Type III (structural failure), Type IV (infection), and Type V (tumor progression) [22].

**Table 1:** Henderson classification [4, 22]

Category	Classification	Failure type	Description
Mechanical	Type I	Soft-tissue failure	Instability, dislocation, tendon rupture
Mechanical	Type II	Aseptic loosening	Clinical and radiological loosening
Mechanical	Type III	Structural failure	Periprosthetic fracture, implant breakage
Non-mechanical	Type IV	Infection	Periprosthetic infection (without retention possibility)
Non-mechanical	Type V	Tumor progression	Tumor recurrence with prosthesis involvement

Infection management was not fully standardized over the study period and was performed according to institutional practice standards and surgeon discretion at the time of treatment, including DAIR, staged revisions, and culture-guided antibiotic therapy. Infection diagnosis was based on the clinical, laboratory, and microbiological criteria applied at the time of treatment. Following publication of the MSIS criteria, elements of these criteria were incorporated into institutional practice; however, formal MSIS scoring was not consistently documented in the medical records throughout the entire study period. Therefore, infection classification in this retrospective analysis reflects contemporaneous clinical decision-making rather than a uniform post hoc application of standardized criteria.

In patients with long-term follow-up, functional outcomes were assessed using three validated scoring systems, representing patient-reported and clinician-based measures of postoperative function. The Toronto Extremity Salvage Score (TESS), The Musculoskeletal Tumor Society (MSTS) and the Oxford Hip Score (OHS).

Statistical analysis was performed using Jamovi, R, and Python. Continuous variables were summarized as medians with interquartile ranges and compared between groups using Mann–Whitney U or Kruskal–Wallis tests, as appropriate. Categorical variables were summarized as frequencies and percentages and compared using Chi-square or Fisher’s exact tests based on cell size.

No formal sensitivity analysis was performed. In Kaplan–Meier analyses, patients were censored at their last documented follow-up. Complication and reoperation events were identified from institutional records and supplemented by telephone follow-up when available. Baseline characteristics were compared between patients lost to follow-up and those retained to assess potential attrition bias.

Associations between continuous variables were assessed using linear regression. Predictors of reoperation were evaluated using binary logistic regression at the procedure level. Variables included in the model were selected a priori based on clinical relevance (revision status, age, ASA score, BMI). Given the limited number of reoperation events, multivariable models were restricted to clinically relevant predictors to reduce the risk of overfitting. Results are reported as odds ratios (OR) with 95% confidence intervals, and statistical significance was defined as *p* < 0.05.

Ethical approval for this retrospective study was obtained from the Ethics Committee of Hannover Medical School. The study was conducted in accordance with the Declaration of Helsinki.

## Results

A total of 81 patients who underwent 84 proximal femoral replacements were included. Patient characteristics are summarized in Table 2. The median age at surgery was 70 years (IQR, 58–78), with a statistically significant difference between primary and revision procedures (*p* = 0.030), as patients aged 70 years or older were more common in the revision group. The cohort included 44.0% male and 56.0% female patients, with no sex differences between groups (*p* = 0.826). Obesity was present in 25.0% of patients (*p* = 0.450), and the median ASA score was 3 across groups (*p* = 0.936). Intensive care admission occurred in 46.4% of procedures and was more frequent following primary PFR (57.1% vs. 35.7%; *p* = 0.049). The median operative time was 197 min (IQR, 151.8–253.8), with longer procedures observed in primary PFR compared with revision PFR (216 vs. 182.5 min), although this did not reach statistical significance (*p* = 0.066). Median follow-up was 7 months (IQR, 0–31), with no significant differences between groups.

**Table 2:** Patient and procedure characteristics according to type of surgery (Primary vs. Revision)

Variable Sociodemographic	Total (*N* = 84)	Primary (*N* = 42)	Revision (*N* = 42)	*p*-value
**Age (years)**				0.030*§
< 50	10 (11.9%)	7 (16.7%)	3 (7.1%)	
50–69	32 (38.1%)	20 (47.6%)	12 (28.6%)	
≥ 70	42 (50.0%)	15 (35.7%)	27 (64.3%)	
**Sex**				0.826
Male	37 (44.0%)	18 (42.9%)	19 (45.2%)	
Female	47 (56.0%)	24 (57.1%)	23 (54.8%)	
**Obesity**				0.450
Yes	21 (25.0%)	9 (21.4%)	12 (28.6%)	
No	63 (75.0%)	33 (78.6%)	30 (71.4%)	
BMI pre-op (Mean ± SD)	26.52 ± 4.86	25.60 ± 4.89	27.45 ± 4.70	0.091
ASA pre-op (Median, IQR)	3.0 (2–3)	3.0 (2–3)	2.5 (2–3)	0.936
ICU stay				0.049*
Yes	39 (46.4%)	24 (57.1%)	15 (35.7%)	
No	45 (53.6%)	18 (42.9%)	27 (64.3%)	
Operative time (min), Median (IQR)	197 (151.8–253.8)	216 (165.8–275.3)	182.5 (145.5–242.0)	0.066
**Vital status**				0.740
Alive	12 (14.3%)	7 (16.7%)	5 (11.9%)	
Dead	24 (28.6%)	13 (31.0%)	11 (26.2%)	
Lost to follow-up	48 (57.1%)	22 (52.3%)	26 (61.9%)	
Follow-up (months), Median (IQR)	7.0 (0–31.0)	5.5 (0–29.3)	10.0 (0–31.0)	0.576
**Complication**				0.008*
Yes	36 (42.9%)	12 (28.6%)	24 (57.1%)	
No	48 (57.1%)	30 (71.4%)	18 (42.9%)	
**Complication classification (Henderson**,** n = 36)**				< 0.001*
Type I – Soft-tissue failure	9 (25.0%)	5 (41.7%)	4 (16.7%)	
Type III – Mechanical failure	7 (19.4%)	1 (8.3%)	6 (25.0%)	
Type IV – Infection	15 (41.7%)	1 (8.3%)	14 (58.3%)	0.005*
Type V – Tumor recurrence	5 (13.9%)	5 (41.7%)	0 (0.0%)	0.002*
**Reoperation (among complications)**				0.134
Yes	24 (66.7%)	6 (50.0%)	18 (75.0%)	
No	12 (33.3%)	6 (50.0%)	6 (25.0%)	

Data are presented as median (IQR), mean ± SD, or n (%). Unless otherwise specified, analyses were performed at the procedure level (N = 84). A total of 81 patients underwent 84 procedures. BMI, body mass index; ASA, American Society of Anesthesiologists physical status classification; ICU, intensive care unit; PFR, proximal femoral replacement. P values were calculated using the Mann–Whitney U test for continuous variables and the chi-square or Fisher’s exact test for categorical variables. Statistical significance was defined as p < 0.05 and is indicated by *

### Complications

**Fig. 1:**
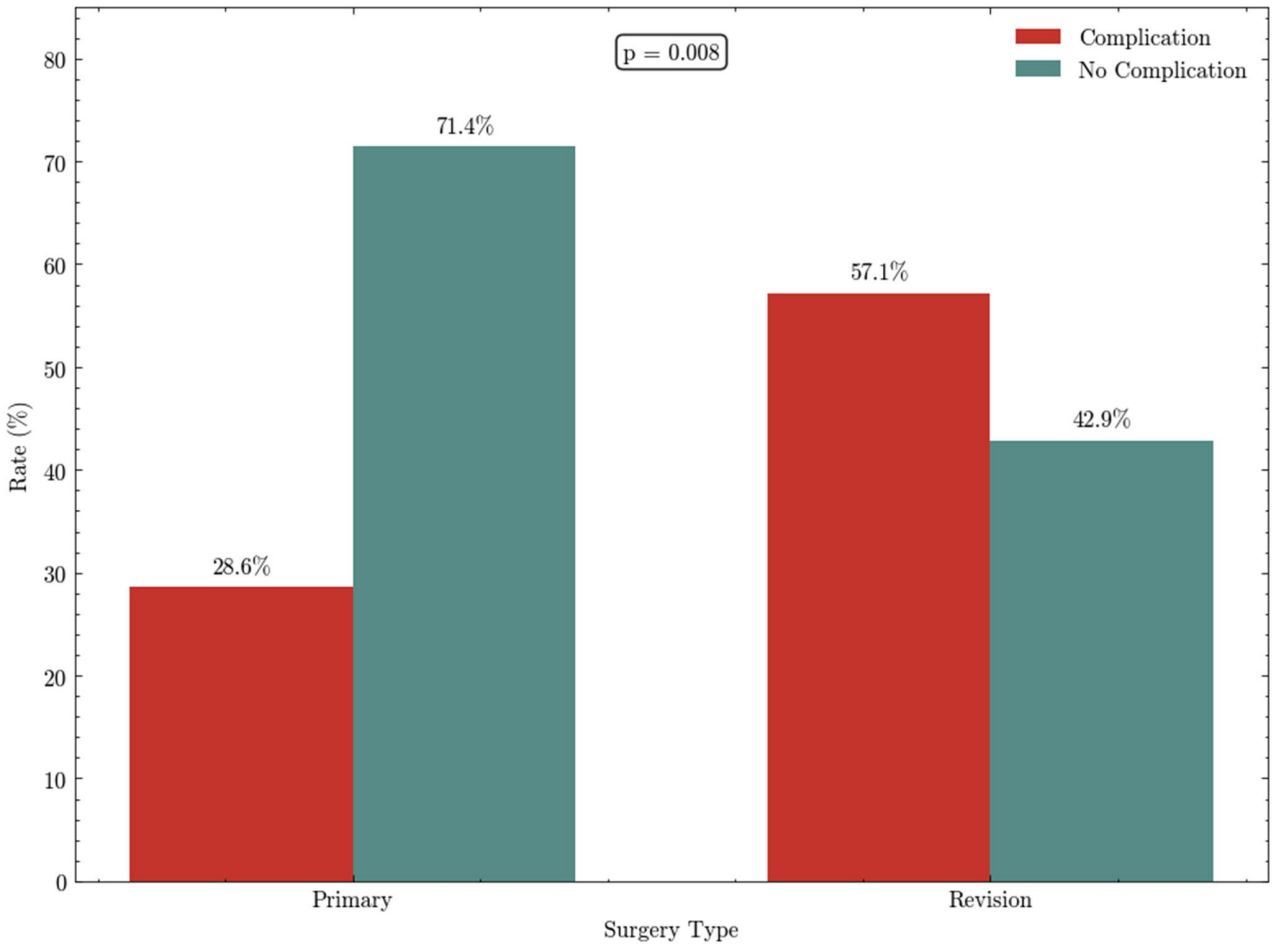
Complication rates in primary versus revision proximal femoral replacement procedures. Primary PFR demonstrated significantly lower complication rates compared with revision PFR (28.6% vs. 57.1%, *p* = 0.008)

Complications occurred in 36 of 84 procedures (42.9%), and the rates were significantly higher after revision PFR (57.1%) compared with primary PFR (28.6%) (*p* = 0.008), as illustrated in Fig. 1. According to the Henderson classification, the distribution of failure modes differed significantly between groups (*p* < 0.001). Soft-tissue failure (Type I) occurred in 9 cases (25.0%), structural failure (Type III) in 7 cases (19.4%), infection (Type IV) in 15 cases (41.7%), and tumor progression (Type V) in 5 cases (13.9%). No aseptic loosening (Type II) was identified in this cohort. Infection was significantly more common in revision procedures, occurring in 58.3% of revision complications compared with 8.3% of primary complications (*p* = 0.005). Microbiological cultures most commonly identified coagulase-negative staphylococci, particularly Staphylococcus epidermidis and Staphylococcus capitis. Methicillin-resistant Staphylococcus aureus (MRSA/ORSA) was documented in isolated cases. Notably, several infections involved Candida albicans, including mixed bacterial–fungal infections, reflecting the complex and often polymicrobial nature of infection following revision PFR. Type V failures occurred exclusively in primary tumor cases (*p* = 0.002). In the tumor group, six patients (14.0%) died within 90 days postoperatively. Four deaths were attributable to tumor progression in the setting of advanced disease, and two to respiratory insufficiency associated with severe underlying comorbidities. In the infection group, one patient (5.0%) died due to respiratory insufficiency and underlying comorbidities.

### Reoperations

Reoperation was required in 24 procedures (28.6%). Among procedures with complications, 66.7% required surgical revision. In unadjusted comparisons, reoperation occurred more frequently after revision PFR (75.0%) than after primary PFR (50.0%), although this difference did not reach statistical significance (*p* = 0.134). Reoperation occurred in 14.0% of tumor procedures (6/43), 65.0% of infection procedures (13/20), and 23.8% of other-indication procedures (5/21). Among cases with complications, reoperation was required in 50.0% of tumor complications (6/12), 86.7% of infection complications (13/15), and 55.6% of other-cause complications (5/9).

In multivariable logistic regression analysis at the procedure level, revision procedures were independently associated with increased odds of reoperation (OR 3.70; 95% CI 1.23–11.09; *p* = 0.020). Age (OR 1.02 per year; 95% CI 0.98–1.06; *p* = 0.329), ASA score (OR 0.71; 95% CI 0.30–1.69; *p* = 0.441), and BMI (OR 1.06 per kg/m²; 95% CI 0.95–1.18; *p* = 0.295) were not independently associated with reoperation.

**Fig. 2:**
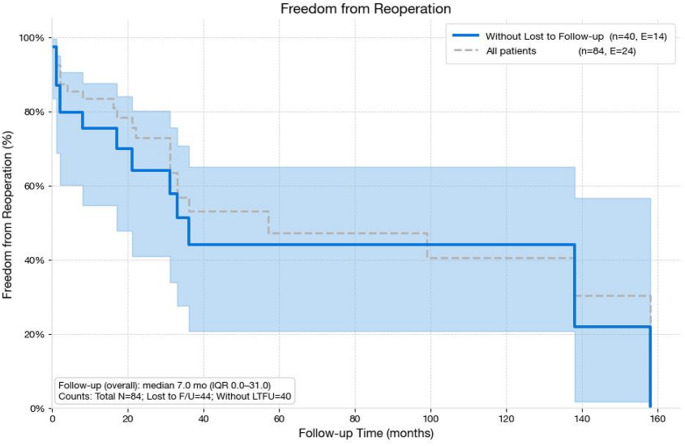
Kaplan–Meier analysis of reoperation-free survival following proximal femoral replacement. The solid blue line represents patients with available follow-up (*n* = 40; events = 14), and the dashed grey line represents the entire cohort (*n* = 84; events = 24). Shaded areas indicate 95% confidence intervals. Median follow-up for the overall cohort was 7.0 months (IQR, 0.0–31.0)

The Kaplan–Meier curve (Fig. 2) demonstrated earlier and steeper decline in reoperation-free survival following revision procedures. Most failures requiring reoperation occurred within the first postoperative year. Four patients ultimately required exarticulation, and one underwent hemipelvectomy due to local tumor recurrence, resulting in an overall limb-salvage rate of 94%. Kaplan–Meier analysis demonstrated a 5-year reoperation-free survival of 68.4% (95% CI, 56.2–80.6%).

A comparison of baseline characteristics between procedures lost to follow-up (*n* = 44) and those retained (*n* = 40) revealed no statistically significant differences in age (*p* = 0.337), ASA score (*p* = 0.772), BMI (*p* = 0.896), procedure type (*p* = 0.827), or indication distribution (*p* = 0.365).

### Outcomes by indication

When stratified by indication, as shown in Table 3, complication rates were 75.0% in infection cases (15/20; 95% CI 50.9–91.3%), 42.9% in other causes (9/21; 95% CI 21.8–66.0%), and 27.9% in tumor-related cases (12/43; 95% CI 15.3–43.7%). Among cases with complications, reoperation was required in 86.7% of infection cases (13/15; 95% CI 59.5–98.3%), 55.6% of other-cause cases (5/9; 95% CI 21.2–86.3%), and 50.0% of tumor cases (6/12; 95% CI 21.1–78.9%). Formal comparisons between indication groups yielded *p* = 0.002 for overall complication rates and *p* = 0.095 for reoperation rates. Given the unequal subgroup sizes and clinical heterogeneity, these analyses should be interpreted cautiously. The estimated 5-year reoperation-free survival of 68.4% in our cohort is comparable to contemporary multicenter series reporting survivorship rates between 60 and 70% at five years, supporting the mid-term durability of modern proximal femoral megaprosthetic reconstruction.

**Table 3:** Results and complications according to surgical indication

Variable	Tumor (*N* = 43)	Infection (*N* = 20)	Other (*N* = 21)	*p*-value
**Age (years)**				0.002*§
< 50	8 (18.6%)	1 (5.0%)	1 (4.8%)	
50–69	22 (51.2%)	7 (35.0%)	3 (14.3%)	
≥ 70	13 (30.2%)	12 (60.0%)	17 (81.0%)	
**Sex**				0.029*%
Male	19 (44.2%)	13 (65.0%)	5 (23.8%)	
Female	24 (55.8%)	7 (35.0%)	16 (76.2%)	
**Operative time (min)**,** Median (IQR)**	217.0 (163.0–312.5)	174.0 (150.0–225.8)	195.0 (138.0–233.0)	0.059
**Follow-up (months)**,** Median (IQR)**	7.0 (0.0–28.5)	18.5 (2.8–33.8)	1.0 (0.0–21.0)	0.113
**90-day mortality**				0.162
Yes	6 (14.0%)	1 (5.0%)	0 (0.0%)	
No	37 (86.0%)	19 (95.0%)	21 (100.0%)	
**Complication (per procedure)**				0.002*
Yes	12 (27.9%)	15 (75.0%)	9 (42.9%)	
No	31 (72.1%)	5 (25.0%)	12 (57.1%)	
**Reoperation (per procedure)**				
Yes	6 (14.0%)	13 (65.0%)	5 (23.8%)	
No	37 (86.0%)	7 (35.0%)	16 (76.2%)	
**Complication classification (Henderson) among complicated cases**	*n* = 12	*n* = 15	*n* = 9	< 0.001*
Type I – Soft-tissue failure	5 (41.7%)	1 (6.7%)	3 (33.3%)	0.126
Type III – Mechanical failure	1 (8.3%)	2 (13.3%)	4 (44.4%)	0.384
Type IV – Infection	1 (8.3%)	12 (80.0%)	2 (22.2%)	0.005*
Type V – Tumor recurrence	5 (41.7%)	0 (0.0%)	0 (0.0%)	0.002*
**Reoperation among complications**				0.095
Yes	6 (50.0%)	13 (86.7%)	5 (55.6%)	
No	6 (50.0%)	2 (13.3%)	4 (44.4%)	

Data are presented as median (IQR) or n (%). Complication and reoperation rates are shown both per procedure (denominator = total procedures per indication) and among complicated cases (denominator = number of complications). Henderson failure mode denominators (e.g., n=12, n=15, n=9) represent the number of complications. P values were calculated using the Kruskal–Wallis test for continuous variables and chi-square or Fisher’s exact tests for categorical variables. Statistical significance was defined as p < 0.05 and is indicated by *. § Significant between Tumor vs. Infection and Tumor vs. Other. % Significant between Infection vs. Other. Type II excluded because no cases occurred

**Fig. 3:**
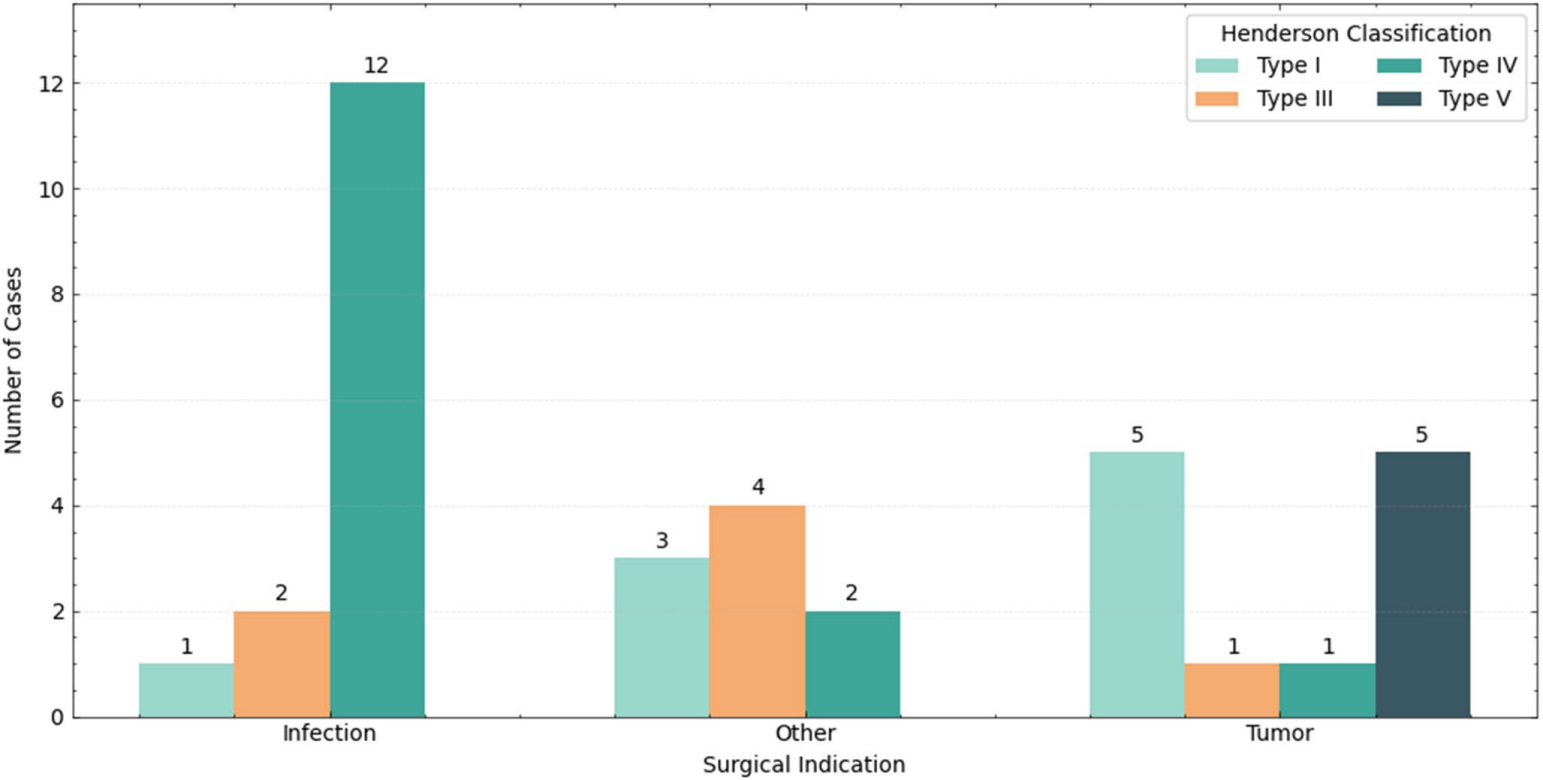
Henderson by indication. Distribution of complications according to the Henderson classification, stratified by surgical indication. Infection cases demonstrated the highest proportion of Type IV failures (*n* = 12), whereas Type V failures occurred exclusively in tumor-related procedures (*n* = 5). Type I and Type III failures were observed across all indications, with the highest Type III failure count occurring in the “Other” group (*n* = 4). No Type II (aseptic loosening) failures were observed in any group

The distribution of Henderson failure types also varied between indications (*p* < 0.001), with results visualized in Fig. 3. Infection cases showed the highest proportion of Type IV failures (80.0%), whereas Type V failures occurred exclusively in tumor cases (41.7%). Operative time was longest in tumor procedures (median, 217 min; IQR, 163.0–312.5), intermediate in other causes (195 min; IQR, 138.0–233.0), and shortest in infection cases (174 min; IQR, 150.0–225.8) (*p* = 0.059). Ninety-day mortality was highest among tumor patients (14.0%), followed by infection cases (5.0%) and none in the other-indication group (*p* = 0.162).

### Extended follow-up functional outcomes

Seven patients had long-term follow-up exceeding 13 years. Functional outcome data were available for these patients only and are presented in Supplementary Table S1. Median scores were 61% for TESS, 56% for MSTS, and 35 for OHS. TESS values were generally the highest, indicating preserved daily function in several patients. MSTS scores showed a wider and overall lower distribution. OHS suggested moderate to good hip-specific function. Despite the small number of long-term survivors, these results demonstrate that acceptable long-term function is achievable after proximal femoral replacement in selected cases. Given the limited sample size, these findings are descriptive and should be interpreted cautiously.

## Discussion

This study evaluated outcomes following proximal femoral replacement across oncologic and non-oncologic indications and demonstrated that revision procedures, particularly those performed for infection, were associated with substantially higher complication and reoperation rates compared with primary PFR, in line with prior literature [7, 9, 10]. Although PFR remains an essential limb-salvage strategy for extensive proximal femoral bone loss, our findings reinforce the considerable morbidity associated with its use in complex revision settings. PFR is one of several reconstructive options for extensive proximal femoral bone loss. Alternatives such as allograft–prosthetic composites, impaction bone grafting with long-stem revision prostheses, and custom 3D-printed implants aim to restore bone stock but are technically demanding and may be associated with graft-related complications, prolonged rehabilitation, or limited availability [16, 20, 23]. In contrast, proximal femoral megaprostheses provide immediate structural stability and early weight-bearing, making them a pragmatic salvage option in cases of massive bone loss or infection, albeit with a substantial complication risk [4, 7, 16, 20, 24].

Consistent with prior literature, infection emerged as the dominant and most challenging failure mode. In our cohort, infection accounted for the majority of complications in revision PFRs and was associated with the highest overall complication and reoperation rates among all indications. These findings align with multicenter analyses reporting infection as the leading cause of failure in megaprosthetic reconstruction, particularly in patients with a history of periprosthetic joint infection or multiple prior surgeries [15, 25]. Given that infection was the predominant failure mode, multimodal prevention strategies are essential. Evidence-based approaches include silver-coated implants in high-risk patients, antibiotic-loaded cement and adherence to optimized perioperative antibiotic prophylaxis protocols [3, 11, 13, 17]. In case of active infection, staged reconstruction with thorough debridement and temporary antibiotic spacers remain the preferred strategy to reduce microbial burden before definitive reimplantation [3, 13, 15]. Adjunctive measures such as local antibiotic carriers or antibacterial surface coatings may further support infection control in selected complex cases [11, 17, 19]. These principles are consistent with contemporary consensus recommendations, including those proposed by the PRO-IMPLANT Foundation [26], although implementation evolved during the study period. The observed infection burden underscores the importance of careful patient selection and optimization of modifiable comorbidities. While silver-coated implants showed a trend toward reduced infection risk, this did not reach statistical significance [11, 17, 19]. Silver-coated components were used selectively during the study period but were not applied in a standardized manner, precluding meaningful subgroup comparison.

The absence of aseptic loosening in this series is noteworthy and consistent with previous observations that modular megaprostheses, especially those used in the proximal femur, exhibit relatively low rates of mechanical loosening compared with other reconstructive sites [2, 6, 20, 27].

In contrast, tumor-related PFR demonstrated comparatively favorable implant-related outcomes, despite the more extensive bone and soft-tissue resection required in oncologic procedures. Complication rates and reoperation rates were lowest in this group; however, these advantages were offset by a higher early mortality rate, reflecting the underlying oncologic burden rather than prosthesis-related factors. These observations are consistent with previous reports in which primary malignant disease, rather than mechanical failure, drives long-term prognosis in tumor patients undergoing PFR [12, 15, 28, 29].

The overall reoperation rate of 29% in this study is similar to reported values in contemporary megaprosthetic literature, where reoperation rates range from 20% to 35% [2, 18]. The distribution of failure modes across indications in our cohort mirrors those reported in prior series, where infection and soft-tissue failure comprise the majority of complications [5, 24, 25]. Mechanical failures (Type III) were observed less frequently but were more common in the non-oncologic group, likely reflecting compromised bone stock, prior implants, and altered biomechanics following earlier surgeries [3, 6, 15]. Soft-tissue failure, particularly abductor insufficiency and postoperative instability, remains a recognized challenge after proximal femoral replacement. In our cohort, soft-tissue reconstruction typically included reattachment of the greater trochanter and abductor mechanism where feasible (e.g., cerclage-based fixation and trochanteric refixation) and reattachment of surrounding musculature using the prosthesis attachment sleeve. In selected complex cases with compromised soft tissues, additional coverage procedures such as rotational muscle flaps were required. Furthermore, acetabular reconstruction strategies such as tripolar or large-head constructs were used selectively to mitigate instability risk in high-risk situations. Despite these measures, soft-tissue failure accounted for 25% of complications in our series, consistent with prior reports identifying instability as a relevant mode of failure following proximal femoral megaprosthetic reconstruction [15, 16, 29].

Given the heterogeneous indications, unequal subgroup sizes, and limited follow-up duration, the results should be interpreted cautiously, as definitive causal inference cannot be established. The heterogeneous indications, variability in prior surgical history, and involvement of multiple surgeons introduce confounding factors that cannot be fully controlled. Detailed microbiological data and standardized infection protocols were not consistently available across the study period. Infection diagnosis and management evolved over time, and the heterogeneity of treatment strategies and surgeon preference may have influenced infection-related outcomes, representing an important limitation. Relevant comorbidities such as diabetes mellitus, immunosuppression, chronic kidney disease, and smoking status are well-established risk factors for infection and adverse outcomes following megaprosthetic reconstruction; however, these variables were not consistently documented [4, 17]. Consequently, they could not be included in the analysis, representing an additional limitation inherent to the retrospective study design. The observed 90-day mortality in the tumor cohort was primarily related to advanced oncologic disease and underlying comorbidities.

Long-term interpretation is limited by substantial loss to follow-up, a recognized challenge in oncologic and elderly multi-morbid populations [17, 28]. Given the 57% loss to follow-up, late complication rates may be underestimated, and true long-term implant survivorship cannot be reliably determined. Functional outcome data were available only for a small subset of long-term survivors, introducing potential survivor bias and limiting generalizability.

Although acceptable function was observed in some patients more than a decade after PFR, functional recovery does not necessarily parallel implant survival, as satisfactory daily activity may persist despite prior complications or limited range of motion [4, 27].

Despite these limitations, this study reflects real-world practice in a high-volume tertiary referral center and represents one of the larger single-center cohorts evaluating both primary and revision PFR using a consistent modular system across oncologic and non-oncologic indications.

Overall, these findings reaffirm that PFR is a reliable salvage procedure for extensive proximal femoral bone loss but carries substantial risk, particularly in the presence of infection and prior revision surgery. Infection remains the principal barrier to durable success and should be the primary focus of perioperative optimization, patient counseling, and future research. Strategies aimed at improving soft-tissue management, reducing microbial load, and optimizing host factors may play a critical role in improving outcomes in this challenging patient population [5, 10, 18, 23, 30, 31].

## Conclusions

Proximal femoral replacement remains an indispensable reconstructive option for patients with extensive proximal femoral bone loss due to oncologic or non-oncologic conditions. This study confirms that while PFR provides reliable mechanical stability and allows limb preservation in situations where conventional reconstruction is not feasible, it is also associated with substantial complication and reoperation rates. Infection continues to represent the most frequent and challenging failure mode, particularly in revision settings, underscoring its dominant role in determining postoperative outcomes. In contrast, primary PFR performed for oncologic indications demonstrated comparatively favorable mechanical outcomes, although these benefits were tempered by the higher early mortality typical of oncologic disease.

No cases of aseptic loosening were observed, supporting previous evidence that modern modular megaprostheses have a low incidence of mechanical loosening. However, the overall burden of complications, especially infection and soft-tissue failure, remains considerable and should be a focal point of preoperative planning and patient counseling. Future research should prioritize strategies aimed at improving infection prevention, optimizing host factors, and reducing the biological challenges associated with revision PFR. Larger multicenter studies with longer follow-up are needed to better evaluate implant survivorship, functional outcomes, and patient-specific risk factors to further refine indications and improve outcomes in this complex patient population.

## Supplementary Information

Below is the link to the electronic supplementary material.

Supplementary Material 1

## Data Availability

No datasets were generated or analysed during the current study.
