# Supplementary material for: Proximal femoral replacement for oncologic and non-oncologic indications: a retrospective study over a 13-year period
Source: Eur J Trauma Emerg Surg. 2026 Mar 31;52(1):116. doi: 10.1007/s00068-026-03151-2 (PMC13038815; doi:10.1007/s00068-026-03151-2)
Supplement: Supplementary file 1 — Supplementary Material 1 [file 68_2026_3151_MOESM1_ESM.docx]

**Supplementary:**

**Table S1.** Individual Long-Term Functional Outcomes (n = 7)

| Patient | TESS (%) | MSTS (%) | OHS (0–48) |
| --- | --- | --- | --- |
| 1 | 71.0 | 70.0 | 35 |
| 2 | 47.0 | 0.0 | 21 |
| 3 | 65.0 | 56.0 | 41 |
| 4 | 61.0 | 87.0 | 45 |
| 5 | 65.0 | 50.0 | 31 |
| 6 | 59.0 | 80.0 | 47 |
| 7 | 37.5 | 33.0 | 33 |

TESS: Toronto Extremity Salvage Score (0–100%, higher scores indicate better function). MSTS: Musculoskeletal Tumor Society Score (0–100%, higher scores indicate better function). OHS: Oxford Hip Score (0–48, higher scores indicate better hip-specific function).
